# Supplementary material for: Feature-specific inhibitory connectivity augments the accuracy of cortical representations
Source: bioRxiv. 2025 Aug 2:2025.08.02.668307. Preprint. [Version 1] doi: 10.1101/2025.08.02.668307 (PMC12324542; doi:10.1101/2025.08.02.668307)
Supplement: 1 [file NIHPP2025.08.02.668307V1-supplement-1.pdf]

bioRxiv preprint doi: https://doi.org/10.1101/2025.08.02.668307; this version posted August 3, 2025. The copyright holder for this preprint (which was not certified by peer review) is the author/funder, who has granted bioRxiv a license to display the preprint in perpetuity. It is made available under aCC-BY 4.0 International license.

Supplementary figure legends:

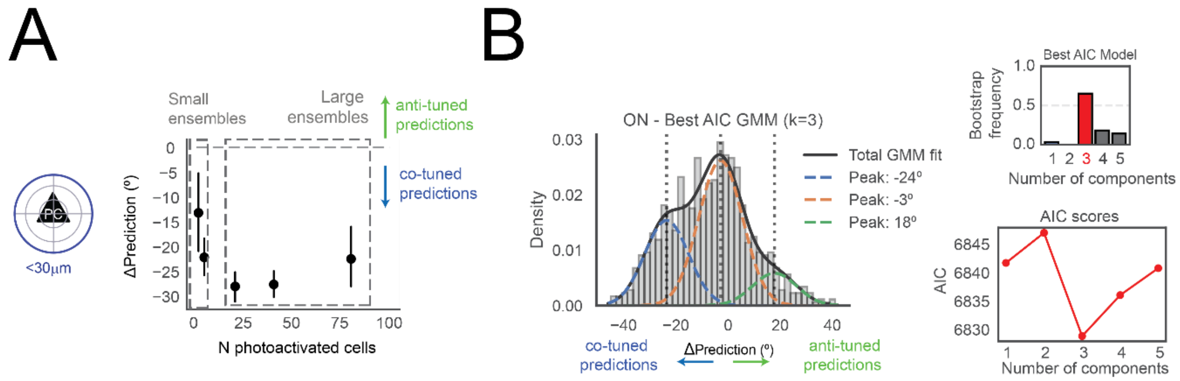

Figure S1. Decoder predictions from targeted and non-targeted neurons

(A) Mean ΔPrediction (°) computed using only the activity of cells within the photostimulated area, shown as a function of ensemble size. All ensembles yielded negative ΔPrediction values, indicating that even small groups of stimulated cells carried sufficient orientation-specific information to bias decoder output toward the target stimulus. Error bars represent 95% confidence intervals across ensembles.

(B) Gaussian Mixture Model (GMM) analysis of ΔPrediction values from the non-targeted responses to all input ensembles. Top left: distribution of ΔPrediction and best-fit 3-component GMM with peaks at approximately –24°, –3°, and +18°, consistent with distinct network regimes (feature completion, neutral, and suppression). Bottom: model comparison using AIC shows that three components consistently provided the best fit. Top right: frequency with which each number of components was selected as the best model across 100 bootstrap replicates.

bioRxiv preprint doi: https://doi.org/10.1101/2025.08.02.668307; this version posted August 3, 2025. The copyright holder for this preprint (which was not certified by peer review) is the author/funder, who has granted bioRxiv a license to display the preprint in perpetuity. It is made available under aCC-BY 4.0 International license.

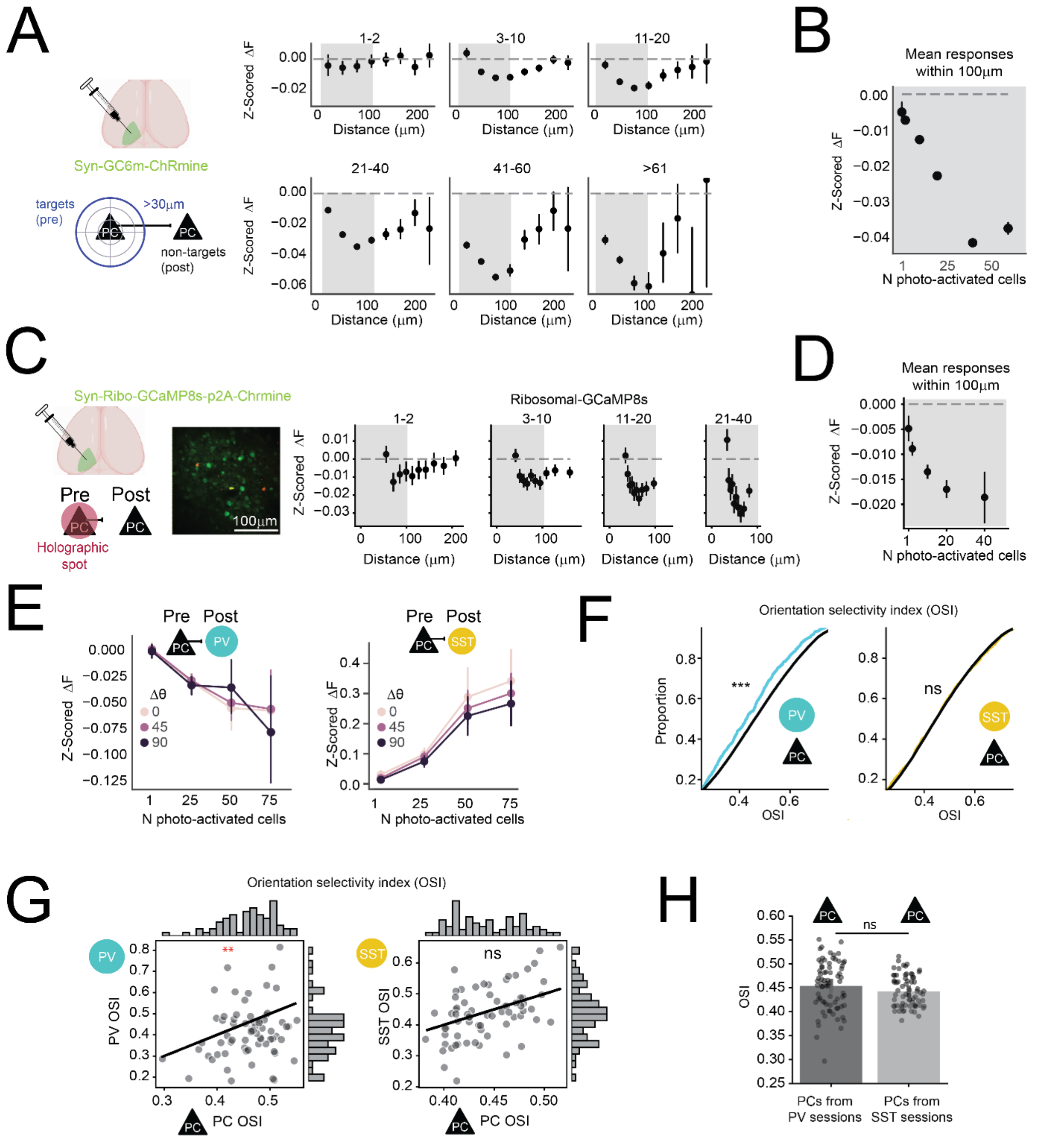

Figure S2. Distance-, size- and tuning-dependent responses of different cell types
(A) Left: Schematic of experimental design. Responses of non-target PCs (>30 μm away) were analyzed as a function of radial distance from the nearest target. Right: Mean z-scored and baseline-subtracted calcium responses (z-scored ΔF) of non-target PCs are plotted across increasing ensemble sizes, grouped by number of photostimulated cells (indicated above each panel). For all ensemble sizes, photostimulation produced distance-dependent suppression of nearby PCs, which gradually recovered at greater distances. Error bars represent 95% confidence intervals across ensembles.

bioRxiv preprint doi: https://doi.org/10.1101/2025.08.02.668307; this version posted August 3, 2025. The copyright holder for this preprint (which was not certified by peer review) is the author/funder, who has granted bioRxiv a license to display the preprint in perpetuity. It is made available under aCC-BY 4.0 International license.

(B) Aggregated mean responses for distances within 100μm from the nearest photostimulated cell for each ensemble size. Error bars represent 95% confidence intervals across ensembles.

(C) Left: Schematic and example image from a mouse expressing ribosomally localized GCaMP8s and Chrmine in PCs. Right: Mean z-scored and baseline-subtracted calcium responses (ΔF) of non-target PCs are plotted across increasing ensemble sizes, grouped by number of photostimulated cells (indicated above each panel).

(D) Aggregated mean responses for distances within 100μm from the nearest photostimulated cell for each ensemble size. Error bars represent 95% confidence intervals across ensembles.

(E) Joint distance- and tuning-dependent responses to PC ensembles in SSTs and PV interneurons. Left: In PV-Cre × Flex-tdTomato mice, photostimulation of PC ensembles suppressed PV interneurons. Right: In SST-Cre × Flex-tdTomato mice SST interneurons showed robust, size-dependent responses both for similarly tuned PCs ($\Delta\theta(PC_{Ens} - SST)$, = 0°, pink), and for anti-tuned PC ensembles ($\theta(PC_{Ens} - SST)$= 90°, purple).

(F) Cumulative distribution of orientation selectivity for PV, SST, and PC populations. PV cells show significantly lower orientation selectivity compared to surrounding PCs (***p < 0.001, Wilcoxon rank-sum test), while SST cells have similar selectivity to PCs (p>0.05). The x axis was cropped between 0.25 and 0.75 for visualization purposes.

(G) Mean orientation selectivity for GABAergic neurons relative to local PCs. Each dot represents a session. Y-axis indicates the mean selectivity of either PVs (left) or SSTs (right). X-axis indicates mean selectivity of PCs. PVs, were reliably less orientation selective compared to nearby PCs (**p= 0.002, Wilcoxon test, N =75 sessions). SSTs were as orientation selective as nearby PCs (p>0.05, Wilcoxon test, N=69 sessions).

(H) Mean orientation selectivity for PCs recorded from PV-tom or SST-tom mice. Each dot represents a session (p>0.05, Wilcoxon Rank Sum test, N>69 sessions).

bioRxiv preprint doi: https://doi.org/10.1101/2025.08.02.668307; this version posted August 3, 2025. The copyright holder for this preprint (which was not certified by peer review) is the author/funder, who has granted bioRxiv a license to display the preprint in perpetuity. It is made available under aCC-BY 4.0 International license.

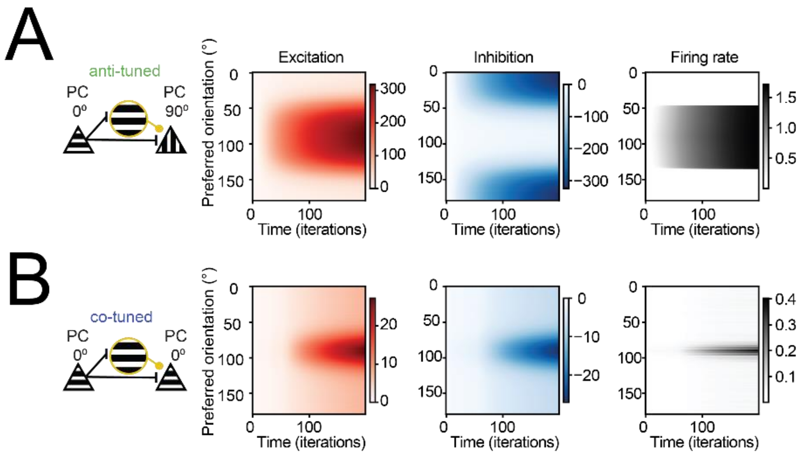

Figure S3. Temporal profiles of excitatory and inhibitory drives in models with opposite inhibitory connectivity

(A) Example simulation showing the evolution of excitation, inhibition, and firing rate for successive iterations within the recurrent loop in response to feedforward input, for a network with anti-tuned inhibition (top row, k_inh < 0). PCs are sorted by their preferred orientation.

(B) Same as in **(A)** but for a model with co-tuned inhibition (k_inh > 0).

bioRxiv preprint doi: https://doi.org/10.1101/2025.08.02.668307; this version posted August 3, 2025. The copyright holder for this preprint (which was not certified by peer review) is the author/funder, who has granted bioRxiv a license to display the preprint in perpetuity. It is made available under aCC-BY 4.0 International license.

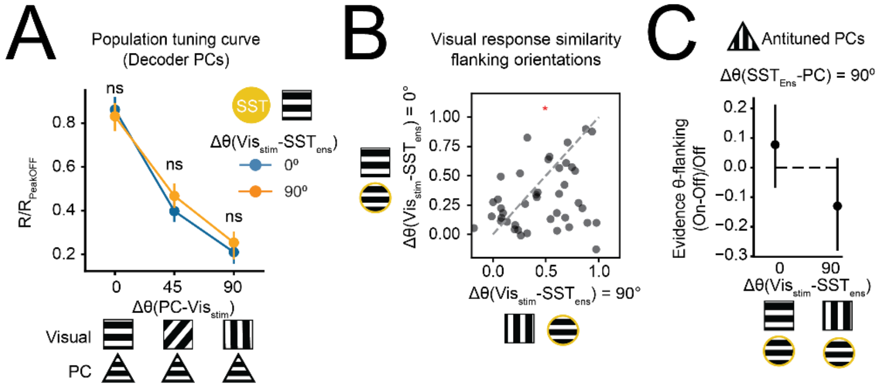

Figure S4. Visual response similarity to flanking orientations in co-tuned and anti-tuned PCs.

(A) Population tuning curves of “decoder-informative” PCs (neurons with greater than zero L1 decoder weights) in SST aligned and SST misaligned trials. No differences were observed across any visual condition (p>0.05 for $\Delta\theta(PC - Vis_{Stim})$ 0°, 45°, or 90°).

(B) Cosine similarity between responses to flanking visual orientations, normalized by the similarity between responses to the same visual orientation. Visually aligned SST ensembles enhance decorrelation of flanking visual responses relative to misaligned ensembles (*p=0.01, Wilcoxon test, N = 39 visual comparisons, 21 ensembles, 9 sessions, 6 mice).

(C) SST activation does not modify the relative evidence for flanking orientations carried by anti-tuned PCs ($\Delta\theta(SST_{Ens} - PC)$= 90°) (p>0.05, for both $\Delta\theta(Vis_{Stim} - SST_{Ens})$ = 0° and 90°, permutation test. N = 23 ensembles, 9 sessions, 6 mice).
